# Mangiferin, an Anti-HIV-1 Agent Targeting Protease and Effective against Resistant Strains

**DOI:** 10.3390/molecules16054264

**Published:** 2011-05-24

**Authors:** Rui-Rui Wang, Yue-Dong Gao, Chun-Hui Ma, Xing-Jie Zhang, Cheng-Gang Huang, Jing-Fei Huang, Yong-Tang Zheng

**Affiliations:** 1 Key Laboratory of Animal Models and Human Disease Mechanisms of the Chinese Academy of Sciences & Yunnan Province, Kunming Institute of Zoology, Chinese Academy of Sciences, Kunming 650223, China; 2 State Key Laboratory of Genetic Resources and Evolution, Kunming Institute of Zoology, Chinese Academy of Sciences, Kunming 650223, China; 3 Graduate School of Chinese Academy of Sciences, Beijing 100039, China; 4 Shanghai Institute of Materia Medica, Chinese Academy of Sciences, Shanghai 201203, China

**Keywords:** mangiferin, HIV-1, protease, anti-HIV agents, drug resistance

## Abstract

The anti-HIV-1 activity of mangiferin was evaluated. Mangiferin can inhibit HIV-1_ⅢB_ induced syncytium formation at non-cytotoxic concentrations, with a 50% effective concentration (EC_50_) at 16.90 μM and a therapeutic index (TI) above 140. Mangiferin also showed good activities in other laboratory-derived strains, clinically isolated strains and resistant HIV-1 strains. Mechanism studies revealed that mangiferin might inhibit the HIV-1 protease, but is still effective against HIV peptidic protease inhibitor resistant strains. A combination of docking and pharmacophore methods clarified possible binding modes of mangiferin in the HIV-1 protease. The pharmacophore model of mangiferin consists of two hydrogen bond donors and two hydrogen bond acceptors. Compared to pharmacophore features found in commercially available drugs, three pharmacophoric elements matched well and one novel pharmacophore element was observed. Moreover, molecular docking analysis demonstrated that the pharmacophoric elements play important roles in binding HIV-1 protease. Mangiferin is a novel nonpeptidic protease inhibitor with an original structure that represents an effective drug development strategy for combating drug resistance.

## 1. Introduction

The development of antiretroviral therapies to fight human immunodeficiency virus type 1 (HIV-1) infection has led to a significant decrease in mortality and morbidity among infected populations. Nevertheless, the emergence of viral resistant strains to drugs is causing serious clinical and public health problems throughout the world. Resistance to one drug often results in cross-resistance to others in the same class [1]. For example, strains highly resistant to one protease inhibitor (PI) are often cross-resistant to other approved PIs [2,3,4]. Some patients die from acquired immune deficiency syndrome (AIDS) caused by multiresistant viruses [5], and the incidence of primary HIV resistance is increasing in various parts of the world [6,7,8,9]. Currently, substantial numbers of HIV-1-infected individuals receiving antiretroviral therapy may harbor viruses broadly cross-resistant to PIs [10]. Consequently, there is an urgent clinical need to develop new PIs that are able to inhibit these broadly resistant HIV-1 variants. At present, there are 10 PIs officially licensed for the treatment of HIV infections. Except for tipranavir (TPV), which is a coumarin derivative, all other compounds can be considered peptidomimetics [11]. TPV is the only approved nonpeptidic protease inhibitor (NPPI) with potent *in vitro* activity against a variety of HIV-1 laboratory strains, clinical isolates and PI-resistant viruses [12]. Structurally, TPV establishes fewer water-mediated hydrogen bonds (H-bonds) compared to other PIs. TPV makes direct H-bonds to the Ile50 residues in the flap instead, while all other PIs bind through water-mediated H-bonds [13]. 

**Figure 1:**
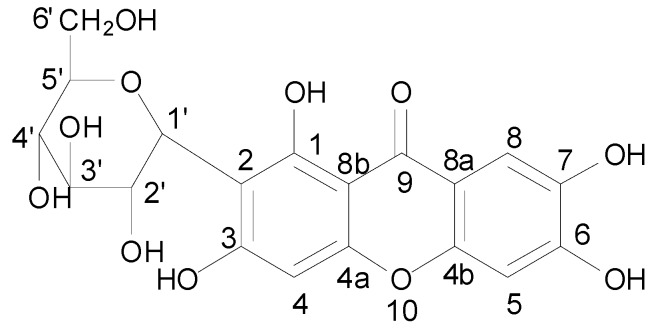
Structure of mangiferin.

Mangiferin (1,3,6,7-tetrahydroxy-C2-β-D-glucoside, Figure 1) is found in many plants, including *Anacardiaceae* and *Rhizoma anemarrhenae*. This chemical is a constituent of folk medicines and can be synthesized easily. The pharmacology of mangiferin is gaining increased attention owing to its modulating effects on oxidative mechanisms in various disorders [14,15,16,17,18]. This compound has also been shown to exhibit antitumor [19], antiviral [20,21,22], immunomodulatory [19,23,24] and radioprotective [18] activities under different experimental conditions. In the present study, mangiferin was confirmed to have good anti-HIV-1 activity. The mechanism of action for mangiferin was then investigated. The results showed that HIV protease (PR) was the chief target. 

Mangiferin is a small compound with a molecular weight of 422. It is interesting that the PR gene mutants HIV-1_RF/V82F/184V_ and HIV-1_L__10R/M46I/L63P/V82T/I84V_ are all mangiferin sensitive. To identify essential chemical features of mangiferin, a pharmacophore model was generated using Discovery Studio 2.0 software (Accelrys, Inc.). The pharmacophore model of mangiferin is described by four pharmacophore elements: two H-bond donors and two H-bond acceptors. A reference pharmacophore model was also extracted from approved PIs. After pharmacophore alignment, three pharmacophore elements of mangiferin displayed a high degree of match with the reference pharmacophore model, and one novel element was obtained. The CDOCKER algorithm [25] was employed to investigate a possible binding manner of mangiferin in HIV-1 PR. These results revealed that the pharmacophore binding characters of mangiferin and the PIs were not completely the same. Because we have already seen inhibitory activities of mangiferin against resistant strains, it represents a novel small molecule PI with exciting prospects for combating drug resistance.

## 2. Results and Discussion

### 2.1. Cytotoxicity of Mangiferin

**Figure 2:**
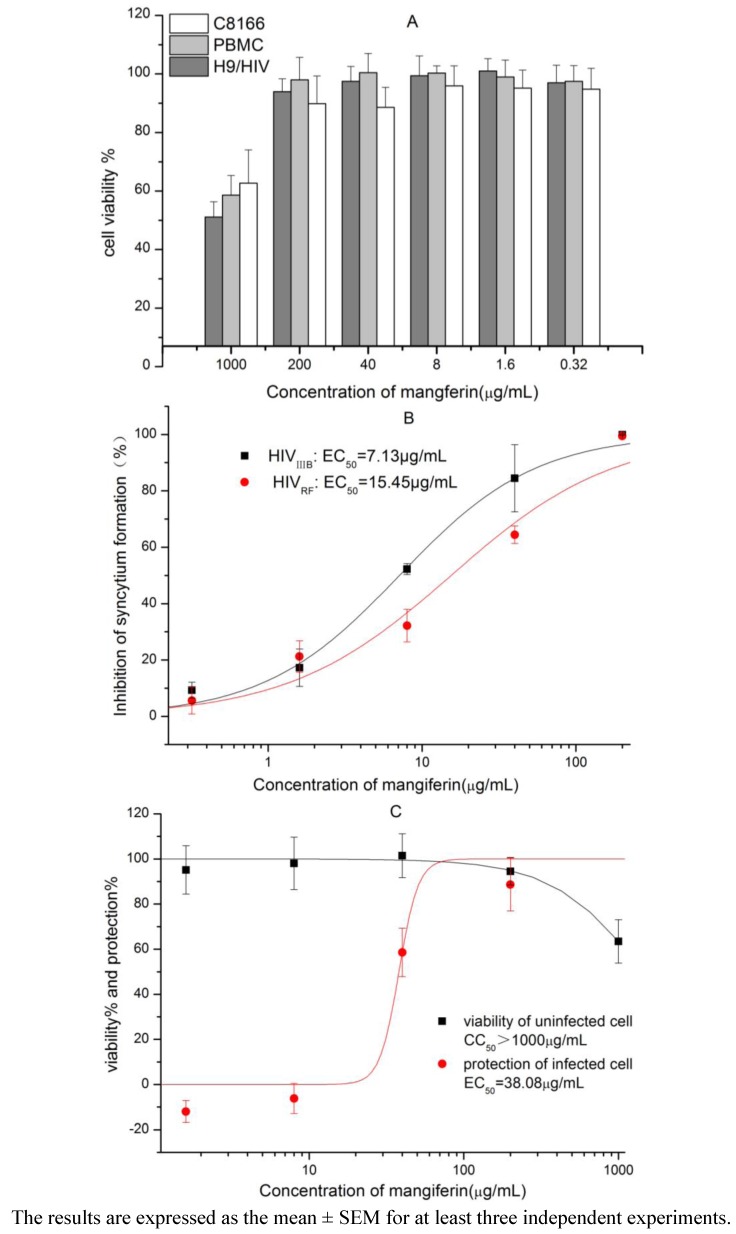
Cytotoxicities of mangiferin on HIV-1 in C8166, PBMC and HIV-1_IIIB_ chronically infected H9 cells (**A**). The inhibition of mangiferin on HIV-1_IIIB _and HIV-1_RF_ replication assessed by syncytium formation (**B**). Protection against lysis and cytotoxicity of mangiferin as determined in mock-infected and HIV-1_IIIB _infected MT-4 cells (**C**).

The effect of mangiferin on the viability of cells was examined (Figure 2A and Figure 2C). The results indicated that this compound exhibited low cytotoxicity on C8166, MT-4, chronically-infected H9 cells and PBMC, and the 50% cytotoxic concentration (CC_50_) values were all above 1,000 μg/mL (2369.67 μM). As control，IDV showed cytotoxicity on C8166 with the CC_50_ value above 500 μM.

### 2.2. Anti-HIV-1 Activity and Mechanism of Mangiferin

The dose-dependent inhibitory activity of mangiferin on HIV-1 induced syncytium formation (cytopathic effects) is shown in Figure 2B. The EC_50_ values of mangiferin in HIV-1_IIIB_ and HIV-1_RF_ for inhibiting cytopathic effects were 7.13 μg/mL (16.90 μM) and 15.45 μg/mL (36.61 μM), respectively. The therapeutic indexes (TI) of this compound were above 140 and 65, respectively. Moreover, the activity of mangiferin in the protection of MT-4 cells from lysis induced by HIV-1_IIIB_ is shown in Figure 2C, in which the TI value was above 26.26. 

The antiviral activities of mangiferin were not limited to prototypic viruses. The EC_50_ values from the p24 antigen assay for the primary HIV-1 isolate HIV-1_KM__018_ and the EC_50_ values from the syncytium assay for the nonnucleoside reverse transcriptase inhibitors (NNRTIs) resistant strain HIV-1_A__17_ were 14.94 μg/mL (35.40 μM) and 9.60 μg/mL (22.75 μM), respectively (Table 1). 

**Table 1:** Inhibitory effects and mechanism of action of mangiferin.

Compounds	(EC_50_, μM)^ a^					
	HIV-1_KM__018_	HIV-1_A__17_	IN activity	RT activity	PR activity	Co-Culture
Mangiferin	35.40 ± 0.02	22.75 ± 3.36	NI	NI	342.80 ± 60.69	NI
AZT	0.90 ± 0.07	0.032 ± 0.008	ND	ND	ND	ND
NVP	ND	0.101 ± 0.038	ND	ND	ND	ND
T-20	ND	ND	ND	ND	ND	0.0058 ± 0.002
IDV	ND	ND	ND	ND	0.19 ± 0.02	ND
PFA	ND	ND	ND	2.45 ± 0.16	ND	ND

^a^ Data represent the average ± standard deviation for at least three independent experiments; NI: no inhibition; ND: not detected; AZT: Zidovudine,nucleoside reverse transcriptase inhibitor; NVP: Nevirapine, nonnucleoside reverse transcriptase inhibitor; T-20: Enfuvirtide, fusion inhibitor; IDV: Indinavir, protease inhibitor; PFA: Foscarnet, RT inhibitor.

**Figure 3:**
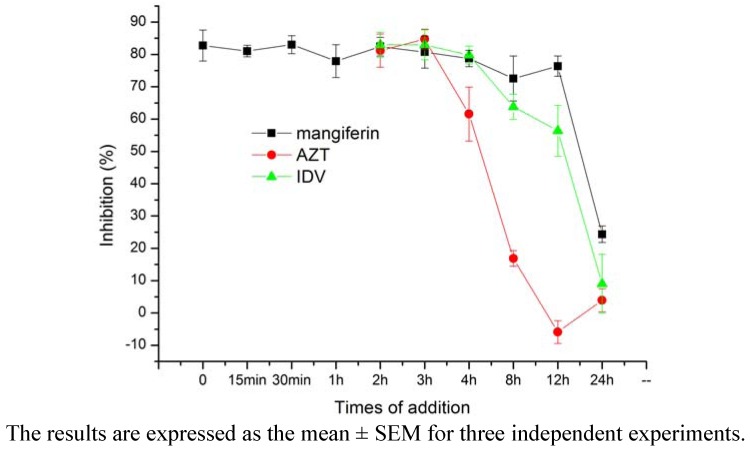
Time-of-addition assay of mangiferin.

We used a time-of-addition assay to determine the stage of the HIV-1 replication cycle with which mangiferin interfered. After HIV-1_IIIB_ infected C8166 cells, mangiferin added at various times blocked p24 antigen production (Figure 3). The results demonstrated that mangiferin might have inhibited HIV-1 PR activity because it blocked HIV-1 p24 antigen production after 12 h. While in a parallel assay, AZT and IDV blocked HIV-1 p24 antigen production after 4 h and 12 h, respectively.

Mechanism studies revealed that mangiferin had no activity on HIV-1 entry, IN or RT, but had some inhibition of HIV-1 PR with an EC_50 _of 144.66 μg/mL (342.80 μM) (Table 1). The results showed that HIV-1 PR might the most important target.

### 2.3. The Sensitivity of Mangiferin to HIV-1 Protease Gene Mutants

**Figure 4:**
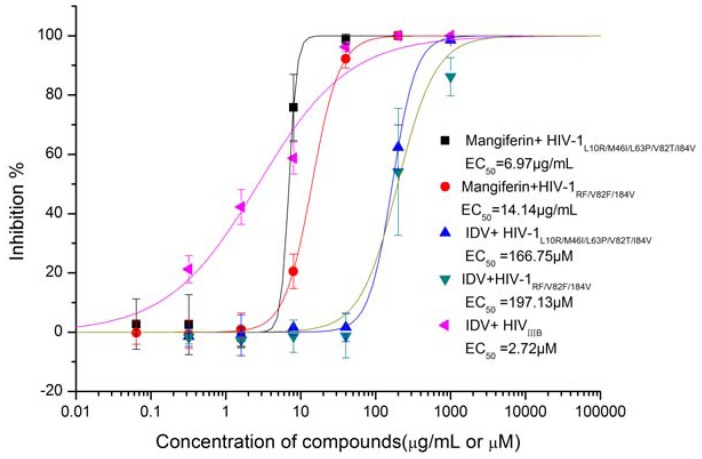
Inhibition of mangiferin and IDV on HIV-1 PR gene mutants.

Two HIV-1 PR gene mutants (HIV-1_L__10R/M46I/L63P/V82T/I84V_ and HIV-1_RF/V82F/184V_) resistant to PIs were used to confirm the action of mangiferin. It was interesting that mangiferin was effective in these two mutants (Figure 4), with EC_50_ values of 6.97 μg/mL (16.52 μM) and 14.14 μg/mL (33.51 μM), respectively. The inhibition activity was similar in HIV-1_IIIB_ and HIV-1_RF_. This was significant, as these viral lines are resistant to conventional chemotherapy (IDV EC_50_ values of 166.75 μM and 197.13 μM, respectively, while the EC_50_ of IDV in wild-type HIV-1_IIIB_ is only 2.72μM). The result implied that mangiferin had different binding mode with IDV. Since IDV was one of the typical PIs, it is of interest to find out the difference.

### 2.4. Pharmacophore Elements of Mangiferin

The pharmacophore model was generated and the top 10 hypotheses were exported. The first hypothesis (Hypo1), was output ranked automatically as the best pharmacophore hypothesis, based on the highest ranking score and good fit values of the inner ranking function in Discovery Studio 2.0 software (Accelrys, Inc.). The pharmacophore models of the reference commercial drugs were described by three pharmacophore elements: two H-bond acceptors (R_HBA2.11, R_HBA3.11) and one H-bond donor (R_HBD1.11). The pharmacophore model of mangiferin contained the same two H-bond acceptors (M_HBA4.11, M_HBA3.11) and one H-bond donor (M_HBD1.11) seen in the reference models. Moreover, one novel H-bond donor (M_HBD2.11) was observed in mangiferin. 

**Figure 5:**
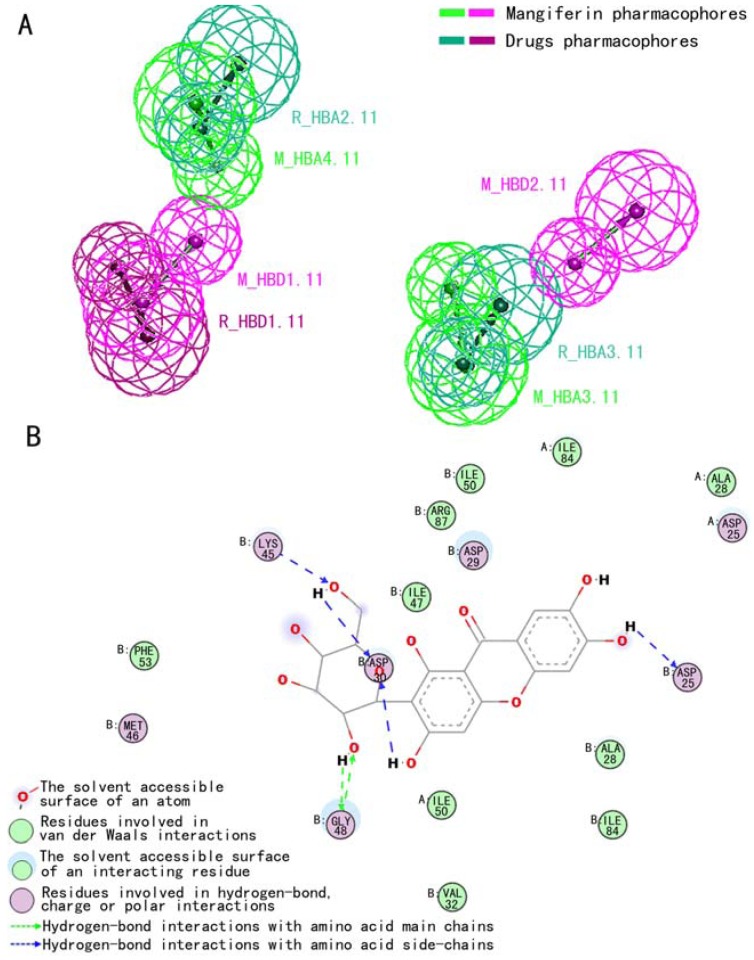
The pharmacophore model alignment between mangiferin and reference drug (**A**). The binding mode of mangiferin in the active site of HIV-1_RF_ PR (**B**).

In the alignment between the pharmacophores of mangiferin and the reference drugs, three pharmacophore elements matched well, with an RMSD value (root mean square deviation) is 2.653673Å between them. The mangiferin pharmacophore elements M_HBA3.11, M_HBA4.11 and M_HBD1.11 corresponded to reference drug pharmacophore elements R_HBA3.11, R_HBA2.11 and R_HBD1.11, respectively (Figure 5A). 

Pharmacophores are a set of structural features in a molecule that are recognized at a receptor site and are responsible for that molecule's biological activity [26]. The pharmacophoric elements exhibited by mangiferin were largely similar to those of drugs currently on the market. Similar pharmacophoric feature is common for molecules with the same biological activity. The similar pattern indicated that mangiferin possessed the basic structural foundation for anti-HIV-1 activity. Pharmacophore model analysis prompted us to investigate the binding model of mangiferin in the HIV-1_RF_ PR complex. 

### 2.5. Binding Mode of Mangiferin in HIV-1 Protease

Ten HIV-1_RF_ mutants were generated, and refinement was carried out at a high optimization level. The protein structures were evaluated using the DOPE [27] (Discrete Optimized Protein Energy) scoring function. The highest DOPE score mutant (the DOPE Score value was -24336.89) was selected for subsequent analysis. From the mangiferin 3D structure, 255 best-quality diverse conformations were generated as a ligand set. Each ligand produced 100 random conformations during docking. The final results exported 400 refined poses. The pose with the highest -CDOCKER_INTERACTION_ ENERGY value (61.2) indicated the most favorable binding and was selected for HIV-1_RF_ PR and mangiferin interaction analysis.

The binding mode of mangiferin in the active site of HIV-1_RF_ PR is shown in Figure 5B. As can be seen, there are many H-bonds between the mangiferin hydroxyl group and various HIV-1_RF_ PR residues in this complex structure. Mangiferin pharmacophoric element M_HBD4.11, corresponding to the 6’-hydroxyl group of the sugar moiety, forms three H-bonds with the amino acid side chain (Asp30, Lys45) of HIV-1_RF_ PR and the 3’-hydroxyl group of mangiferin. Moreover, the 2’-hydroxyl group of mangiferin’s sugar moiety interacts with the amino acid main chain (Gly48) of HIV-1_RF_ PR through H-bonds. The novel pharmacophoric element was observed to play a key role in binding HIV-1_RF_ PR: the 6-hydroxyl group of mangiferin forms a hydrogen bond with the catalytic residue Asp25. Furthermore, other interactions were also observed in the complex structure, including charged, polar and van der Waals interactions (Figure 5B).

Docking results showed that the modeled pharmacophore hydrogen bonding groups located in the ‘head’ and ‘tail’ of mangiferin interacted with HIV-1_RF_ PR. Furthermore, novel pharmacophoric features were observed in mangiferin that played a key role in binding HIV-1_RF_ PR catalytic residue Asp25 and may offer clues to understanding its effectiveness against resistant strains.

## 3. Experimental

### 3.1. Mangiferin Extraction and Separation

Raw roots and rhizomes of *Anemarrhena asphodeloides* Bge*.* (5.0 kg) were extracted with 80% ethanol (25 L × 3, 3 h each time) under reflux. The filtrate was then fractionated with *n*-pentanol (3.5 L × 4) to give fraction A (5 g). This fraction was chromatographed over Sephadex LH-20 (150 g) using MeOH-H_2_O (9:1) as eluent. Forty fractions were collected (40 mL each) and combined according to TLC analysis. Fractions 5-17 gave mangiferin (170 mg). The purity of the mangiferin determined by HPLC was over 95% [28].

### 3.2. Reagents and Chemicals

AZT, HEPES (*N*-2-(2-hydroxyothyl) piperazine-*N′*-(2-ethanesufonic acid)), IDV, MTT (3-(4,5-dimethylthiazol-2-yl)-2,5-diphenyltetrazolium bromide), DMF (*N,N′*-dimethylformamide), PHA (phytohemagglutinin) and PFA were all purchased from Sigma. NVP was purchased from the United States Pharmacopeial Convention Inc. T-20 was purchased from Merck.

### 3.3. Cells and Viruses

Human T-cell lines (C8166, MT-4) and chronically infected H9/HIV-1_IIIB_ were kindly donated by the Medical Research Council (MRC), the AIDS Reagent Project, U.K. All of the cell lines and viruses were maintained at 37 °C under 5% CO_2_ in RPMI-1640 medium (Gibco) supplemented with 10% (v/v) heat-inactivated newborn calf serum (NCS). Peripheral blood mononuclear cells (PBMCs) from healthy donors were isolated by Ficoll–Hypaque centrifugation and incubated in complete medium containing 5 μg/mL PHA for 72 h prior to use in antiviral assays. 

The laboratory-derived strains HIV-1_IIIB_ and HIV-1_RF_, The NNRTIs resistant strain HIV-1_A__17_*,* PR Gene Mutants HIV-1_RF/V82F/184V_ and HIV-1_L__10R/M46I/L63P/V82T/I84V_ were kindly donated by NIH. The clinically isolated strain HIV-1_KM__018 _was obtained from a naïve HIV-1 infected individual in Yunnan province of China. The 50% HIV-1 tissue culture infectious dose (TCID_50_) in C8166 cells was calculated by the Reed and Muench method. Virus stocks were stored in small aliquots at −70 °C. 

### 3.4. Cytotoxicity Assay

The cytotoxicity of the compounds on C8166 cells was determined by a MTT colorimetric assay as described previously [29]. Aliquots of 100 μL/well (4 × 10^5^/mL) C8166 cell suspension were seeded on a microtiter plate. Next, 100 μL/well of various concentrations of compounds was added and the plates were incubated at 37 °C and 5% CO_2_ for 3 days. The MTT reagent was added and incubated for 4 h, 100 μL of supernatant was discarded and then 100 μL of 50% DMF–20% SDS was added. The plates were read on an ELISA reader (Elx800, Bio-Tek) at 595/630 nm. The CC_50_ was calculated from the dose response curve. 

### 3.5. Antiviral Activity Against Acute HIV-1 Infection

The inhibitory activities of mangiferin against the HIV-1_ⅢB_ and HIV-1_RF_, HIV-1_KM__018_, HIV-1_A__17_, HIV-1_L__10R/M46I/L63P/V82T/I84V_ and HIV-1_RF/V82F/184V_ were determined as previously described [29,30,31]. Briefly, 4 × 10^4^ C8166 cells were infected with different HIV isolates at a multiplicity of infection (M.O.I.) of 0.06–0.1 for 2–4 h. Then the plates were incubated in the presence or absence of 100 μL of various concentrations of mangiferin. NVP, IDV and AZT were used as positive drug controls. After 3–7 days of culture, the percentage inhibition of syncytia formation was scored or the level of p24 was measured by ELISA and the EC_50_ was calculated. 

### 3.6. Protection for HIV-1 Induced Lytic Effects

The activities of the compound against acute HIV-1 infection were based on the inhibition of HIV-1 induced cytopathogenicity in MT-4 cells, as described previously [31]. Uninfected or HIV-1_IIIB_-infected (MOI = 0.1) MT-4 cells (3 × 10^5^ cells/mL) were seeded in microtiter plates with 100 μL of different concentrations of mangiferin. AZT was used as the control drug. After a 7-day incubation, the viability of both HIV-1 and mock-infected cells were assessed using the MTT method. 

### 3.7. Co-culture Assay

Cell-to-cell fusion between normal C8166 cells and H9 cells chronically infected withHIV-1_IIIB_ was quantified under an inverted microscope. 3 × 10^4^ C8166 cells were co-cultured with 1 × 10^4^ H9/HIV-1_IIIB_ cells in the presence or absence of mangiferin at varying serial concentrations. T-20 was used as positive drug control. After an 8 h incubation, the number of syncytia was counted under an inverted microscope [31].

### 3.8. Time of Addition Assay

To determine the stage of the HIV replication cycle with which this anti-HIV compound interfered, a time-of-addition experiment was carried out. C8166 cells were exposed to HIV at M.O.I of 0.2. To ensure that the virus replication steps were synchronized in the whole-cell population, infected cells were incubated for 2 h at 4 °C. After allowing adequate time for adsorption, the unabsorbed virus was removed by washing twice with complete medium. The temperature was then shifted to 37 °C, and mangiferin was added at different times (0, 2, 4, 6, 8, 12, 24 and 48 h) after adsorption. AZT and IDV were used as positive drug control. After 3 days of culture, HIV-1 p24 expression was detected by ELISA. 

### 3.9. HIV Reverse Transcriptase, Integrase and Protease Assay

HIV-1 reverse transcriptase (RT) activity was measured by ELISA using a commercially available kit (Roche) according to the instructions of the manufacturer [30]. PFA was used as positive drug control. The interaction between compounds and HIV-1 integrase (IN) was determined by SPR biosensor technology using a BIAcore 3000 biosensor system (Biacore Inc., Piscataway, NJ). HIV-1 IN were immobilized on the surface of the chip, and 200 μg/mL of compounds diluted in HBS-EP was applied. Dissociation of compounds from the IN was monitored after washing the chip and the kinetic rate constants for dissociation (Kd) was obtained by fitting the real-time data using BIA evaluation software [32]. 

The recombinant HIV-1 PR was expressed and purified as previously described [33]. HIV-1 PR was diluted in reaction buffer and the compounds were added and incubated for 30 min at room temperature. Fluorescent substrate DABCYL-γ-Abu-Ser-Gln-Asn-Tyr-Pro-Ile-Val-Gln- EDANS (Anaspec, San Jose) was added to initiate the reaction. The mixture was allowed to react for 90 min and the change of the fluorescent signal was monitored. IDV was used as positive drug control. The percentage of inhibition was calculated from the change in fluorescence.

### 3.10. Pharmacophore Model Analysis

Three-dimensional structures of mangiferin and 10 commercial drugs from the ACD3D database (Symyx, Inc.) were imported into Catalyst [34], wherein subsequent pharmacophore model generation was performed. The reference compounds included the FDA-approved drugs APV (amprenavir), ATV (atazanavir), TMC-114(darunavir), Fos-APV (fosamprenavir), IDV, lopinavir, NFV (nelfinavir), RTV (ritonavir), Saquinavir and TPV. The maximum of 255 conformers were generated using the BEST conformation generation method [35] to ensure the best coverage of conformational space. The pharmacophore features included were hydrogen bond acceptors, hydrogen bond donors, and hydrophobes. 

The reference pharmacophore features were generated based on ten drugs currently on the market and were verified by virtual screening of the database, which consists of diverse conformers from 10 commercially available drugs. Using the HipHop algorithm [36] internal scoring function, the best pharmacophores were output first. To find a consensus pharmacophore or distinguishable feature, the Pharmacophore Comparison Protocol aligned mangiferin’s pharmacophores to reference pharmacophores. 

### 3.11. Molecular docking

Using the known structure as a template (PDB code: 3D3T), several mutants were constructed from its sequence by MODELER [37] to generate the HIV-1_RF_ structure. The MODELER uses different scoring functions and optimization protocols [38] to optimize all the atoms of the mutated residues. The structures of mangiferin came from the ACD3D database (Symyx, Inc.). Afterwards, best-quality diverse mangiferin conformations were generation to ensure the best coverage of conformational space. The set of diverse conformers of mangiferin was used for docking into the HIV-1_RF_ active pocket. Docking was performed using CDOCKER [25]. The models were energy minimized with the CHARMm force field before performing docking. CDOCKER reports scores based on internal ligand strain energy and receptor-ligand interaction energy. All docking and pharmacophore analysis were performed using Discovery Studio 2.0 software (Accelrys, Inc.).

## 4. Conclusions

Mangiferin exhibited low cytotoxicity and good inhibitory activity on HIV-1 replication in a dose-dependent manner. The antiviral activities of mangiferin were observed to primary HIV-1 isolate and resistant strains also. Mechanism studies revealed that mangiferin might inhibit the HIV-1 protease, and is still effective against HIV peptidic protease inhibitor resistant strains. Furthermore, three pharmacophore elements of mangiferin displayed a high degree of matching with the reference pharmacophore model from approved PIs and as a key role in binding HIV-1_RF_ PR. Though the anti-HIV-1 activities were not as good as approved PIs, our results suggest that mangiferin may be a novel NPPI with an original structure that represents an effective drug development strategy for combating drug resistance.

## References

[1] Deeks S.G., Wrin T., Liegler T., Hoh R., Hayden M., Barbour J.D., Hellmann N.S., Petropoulos C.J., McCune J.M., Hellerstein M.K., Grant R.M. (2001). Virologic and immunologic consequences of discontinuing combination antiretroviral-drug therapy in HIV-infected patients with detectable viremia. N. Engl. J. Med..

[2] Condra J.H., Schleif W.A., Blahy O.M., Gabryelski L.J., Graham D.J., Quintero J.C., Rhodes A., Robbins H.L., Roth E., Shivaprakash M. (1995). *In vivo* emergence of HIV-1 variants resistant to multiple protease inhibitors. Nature.

[3] Gulnik S.V., Suvorov L.I., Liu B., Yu B., Anderson B., Mitsuya H., Erickson J.W. (1995). Kinetic characterization and cross-resistance patterns of HIV-1 protease mutants selected under drug pressure. Biochemistry.

[4] Tisdale M., Myers R.E., Maschera B., Parry N.R., Oliver N.M., Blair E.D. (1995). Cross-resistance analysis of human immunodeficiency virus type 1 variants individually selected for resistance to five different protease inhibitors. Antimicrob. Agents Chemother..

[5] Markowitz M., Mohri H., Mehandru S., Shet A., Berry L., Kalyanaraman R., Kim A., Chung C., Jean-Pierre P., Horowitz A. (2005). Infection with multidrug resistant, dual-tropic HIV-1 and rapid progression to AIDS: a case report. Lancet.

[6] Clavel F., Hance A.J. (2004). HIV drug resistance. N. Engl. J. Med..

[7] Deeks S.G. (2003). Treatment of antiretroviral-drug-resistant HIV-1 infection. Lancet.

[8] Little S.J., Holte S., Routy J.P., Daar E.S., Markowitz M., Collier A.C., Koup R.A., Mellors J.W., Connick E., Conway B. (2002). Antiretroviral-drug resistance among patients recently infected with HIV. N. Engl. J. Med..

[9] Yeni P. (2006). Update on HAART in HIV. J. Hepatol..

[10] Boden D., Markowitz M. (1998). Resistance to human immunodeficiency virus type 1 protease inhibitors. Antimicrob. Agents Chemother..

[11] De Clercq E. (2009). Looking back in 2009 at the dawning of antiviral therapy now 50 years ago an historical perspective. Adv. Virus Res..

[12] Poppe S.M., Slade D.E., Chong K.T., Hinshaw R.R., Pagano P.J., Markowitz M., Ho D.D., Mo H., Gorman R.R., Dueweke T.J. (1997). Antiviral activity of the dihydropyrone PNU-140690, a new nonpeptidic human immunodeficiency virus protease inhibitor. Antimicrob. Agents Chemother..

[13] Muzammil S., Armstrong A.A., Kang L.W., Jakalian A., Bonneau P.R., Schmelmer V., Amzel L.M., Freire E. (2007). Unique thermodynamic response of tipranavir to human immunodeficiency virus type 1 protease drug resistance mutations. J. Virol..

[14] Sanchez G.M., Re L., Giuliani A., Nunez-Selles A.J., Davison G.P., Leon-Fernandez O.S. (2000). Protective effects of Mangifera indica L. extract, mangiferin and selected antioxidants against TPA-induced biomolecules oxidation and peritoneal macrophage activation in mice. Pharmacol. Res..

[15] Muruganandan S., Gupta S., Kataria M., Lal J., Gupta P.K. (2002). Mangiferin protects the streptozotocin-induced oxidative damage to cardiac and renal tissues in rats. Toxicology.

[16] Garrido G., Delgado R., Lemus Y., Rodriguez J., Garcia D., Nunez-Selles A.J. (2004). Protection against septic shock and suppression of tumor necrosis factor alpha and nitric oxide production on macrophages and microglia by a standard aqueous extract of Mangifera indica L. (VIMANG). Role of mangiferin isolated from the extract. Pharmacol. Res..

[17] Andreu G.P., Delgado R., Velho J.A., Curti C., Vercesi A.E. (2005). Iron complexing activity of mangiferin, a naturally occurring glucosylxanthone, inhibits mitochondrial lipid peroxidation induced by Fe2+-citrate. Eur. J. Pharmacol..

[18] Jagetia G.C., Baliga M.S. (2005). Radioprotection by mangiferin in DBAxC57BL mice: A preliminary study. Phytomedicine.

[19] Guha S., Ghosal S., Chattopadhyay U. (1996). Antitumor, immunomodulatory and anti-HIV effect of mangiferin, a naturally occurring glucosylxanthone. Chemotherapy.

[20] Zheng M.S., Lu Z.Y. (1990). Antiviral effect of mangiferin and isomangiferin on herpes simplex virus. Chin. Med. J. (Engl.).

[21] Zhu X.M., Song J.X., Huang Z.Z., Wu Y.M., Yu M.J. (1993). Antiviral activity of mangiferin against herpes simplex virus type 2 in vitro. Zhongguo Yao Li Xue Bao.

[22] Yoosook C., Bunyapraphatsara N., Boonyakiat Y., Kantasuk C. (2000). Anti-herpes simplex virus activities of crude water extracts of Thai medicinal plants. Phytomedicine.

[23] Makare N., Bodhankar S., Rangari V. (2001). Immunomodulatory activity of alcoholic extract of Mangifera indica L. in mice. J. Ethnopharmacol..

[24] Leiro J., Arranz J.A., Yanez M., Ubeira F.M., Sanmartin M.L., Orallo F. (2004). Expression profiles of genes involved in the mouse nuclear factor-kappa B signal transduction pathway are modulated by mangiferin. Int. Immunopharmacol..

[25] Wu G., Robertson D.H., Brooks C.L., Vieth M. (2003). Detailed analysis of grid-based molecular docking: A case study of CDOCKER-A CHARMm-based MD docking algorithm. J. Comput. Chem..

[26] Gund P. (1977). Three-dimensional pharmacophoric pattern searching. Prog. Mol. Subcell Biol..

[27] Shen M.Y., Sali A. (2006). Statistical potential for assessment and prediction of protein structures. Protein Sci..

[28] Miura I., Hostettman K., Nakanishi K. (1978). 13-C-NMR of naturally occurring xanthone aglycones and glycosides. Nouv. J. Chim..

[29] Wang R.R., Gu Q., Wang Y.H., Zhang X.M., Yang L.M., Zhou J., Chen J.J., Zheng Y.T. (2008). Anti-HIV-1 activities of compounds isolated from the medicinal plant Rhus chinensis. J. Ethnopharmacol..

[30] Wang Q., Wang Y.T., Pu S.P., Zheng Y.T. (2004). Zinc coupling potentiates anti-HIV-1 activity of baicalin. Biochem. Biophys. Res. Commun..

[31] Wang R.R., Yang L.M., Wang Y.H., Pang W., Tam S.C., Tien P., Zheng Y.T. (2009). Sifuvirtide, a potent HIV fusion inhibitor peptide. Biochem. Biophys. Res. Commun..

[32] Wang Y.H., Tang J.G., Wang R.R., Yang L.M., Dong Z.J., Du L., Shen X., Liu J.K., Zheng Y.T. (2007). Flazinamide, a novel beta-carboline compound with anti-HIV actions. Biochem. Biophys. Res. Commun..

[33] Wang Y.H., Wang R.R., Yang L.M., Li J.F., Xu W.M., Zheng Y.T. (2006). Expression and Purification of HIV-1 Protease and the Establishment of a Method for Protease Inhibitor Screening. Virol. Sin..

[34] Kirchmair J., Laggner C., Wolber G., Langer T. (2005). Comparative analysis of protein-bound ligand conformations with respect to catalyst's conformational space subsampling algorithms. J. Chem. Inf. Model..

[35] Smellie A., Teig S.L., Towbin P. (1995). Poling: Promoting Conformational Variation. J. Comp. Chem..

[36] Barnum D., Greene J., Smellie A., Sprague P. (1996). Identification of common functional configurations among molecules. J. Chem. Inf. Comput. Sci..

[37] Eswar N., Webb B., Marti-Renom M.A., Madhusudhan M.S., Eramian D., Shen M.Y., Pieper U., Sali A. (2006). Comparative protein structure modeling using Modeller. Curr. Protoc. Bioinformatics.

[38] Feyfant E., Sali A., Fiser A. (2007). Modeling mutations in protein structures. Protein Sci..

